# Recognising and managing bilateral cataracts in children

**Published:** 2024-02-09

**Authors:** Sarah Sitati

**Affiliations:** 1 Consultant Ophthalmologist, Paediatric Ophthalmology & Strabismus, Kenyatta National Hospital, Kenya. Chair, ROP Working Group Kenya.

**Children may be born with cataracts in both eyes (congenital cataract) or the cataracts may become apparent during the first few years of life. These children need high quality cataract surgery as soon as possible, with good follow-up.**

**Figure:**
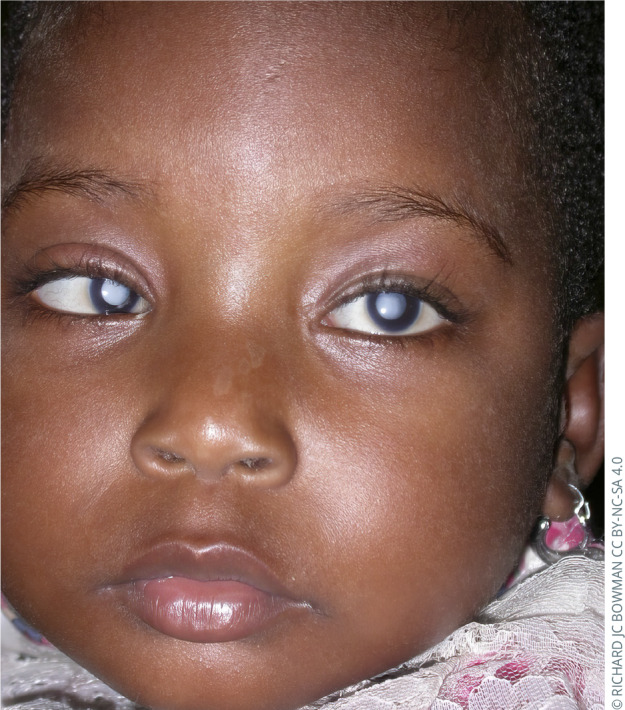
A child with dense, bilateral cataract. Both eyes are likely to be amblyopic if the cataracts were present since birth. tanzania

In many low- and middle-income countries, where it often takes a long time before children receive the surgery they need, children who are born with bilateral cataract may remain severely visually impaired after the cataracts have been surgically removed. This is because, in children, dense cataracts need to be operated on as soon as possible, otherwise the part of the brain responsible for visual processing will not develop normally. If cataract surgery happens too late, vision will be blurred or greatly reduced – known as amblyopia, or ‘lazy eye’.

Even in children in high-income settings, where bilateral congenital cataracts are detected early and surgery is performed promptly (before 3 months of age), the average corrected visual acuity after surgery is 6/12 (logMAR 0.34).^1^ Surgery performed later than 3 months of age will have even poorer outcomes.

Counselling of parents is extremely important at all stages of the child's management, so that they understand why surgery is needed, what will be involved, and what they should expect after surgery. They also need to understand that surgery in children is unlike cataract surgery in adults, which they may have some experience of, and that long term follow-up is of vital importance.

## Causes

Bilateral congenital cataracts are often due to genetic abnormalities,^2^ but the family history is only positive in approximately 20% of cases. Infective causes, which are unusual in most countries, include congenital rubella syndrome and congenital cytomegalovirus infection. Children with these congenital infections may also have systemic problems, such as heart defects, which have implications for general anaesthesia. Bilateral cataracts may also be associated with other eye anomalies, such as microphthalmos.

Acquired causes of bilateral cataract include topical or systemic steroid use, chronic uveitis, certain drugs, and occasionally trauma. Cataracts in older children can be associated with systemic conditions such as diabetes, or atopic conditions such as eczema.

## What to ask about and look for at community or primary level

Babies may present because the mother has noticed something white in the eyes of her child, or an older sibling may have cataracts. In children aged two and above, the carers may notice abnormal visual behaviour: for example, the child does not reach out for or pick up objects, or bumps into (or falls) over things. The carer may also notice that the child has ‘wobbly eyes’ (nystagmus). Older children may have difficulty reading or problems identifying faces and objects. An abnormal red (or fundal) reflex – which may be noted in photographs – can be noticed at any age.

Cataracts can be detected in clinics in the community (at primary level) by testing the child's fundal reflex. For more information, see the article on screening in this issue.

## Examination in the hospital eye department

It is important to take a **history** of when the signs were first noticed and ask about a family history of cataract in childhood, past medical history, and systemic history, including whether steroids have been used.

Look to see whether **nystagmus** is present. If so, this means that the child's vison has been very poor from a very young age and their chances of having good visual acuity after surgery are not very good. However, even if their visual acuity is low, the child will have a better field of vision after surgery; this will increase their independence.

**Figure:**
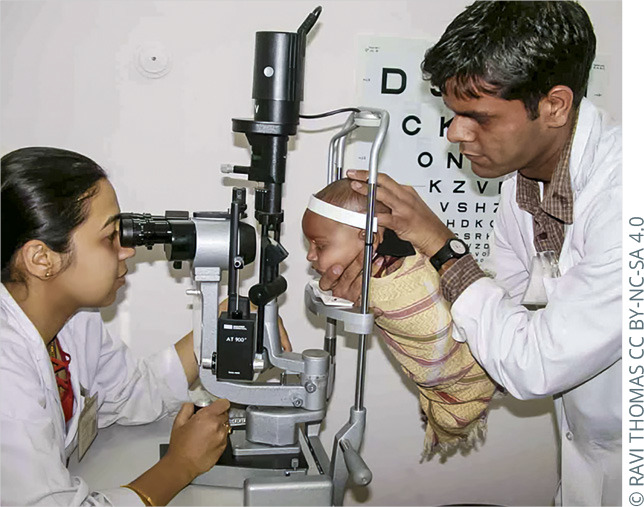
Examining a baby at the slit lamp. india

Cataracts can sometimes complicate **retinal conditions** such as total retinal detachment or retinal dystrophies, which means that surgery will not benefit the child. Examine the pupil reactions and test light projection. B-scan ultrasound can also be useful.

**Figure 1:**
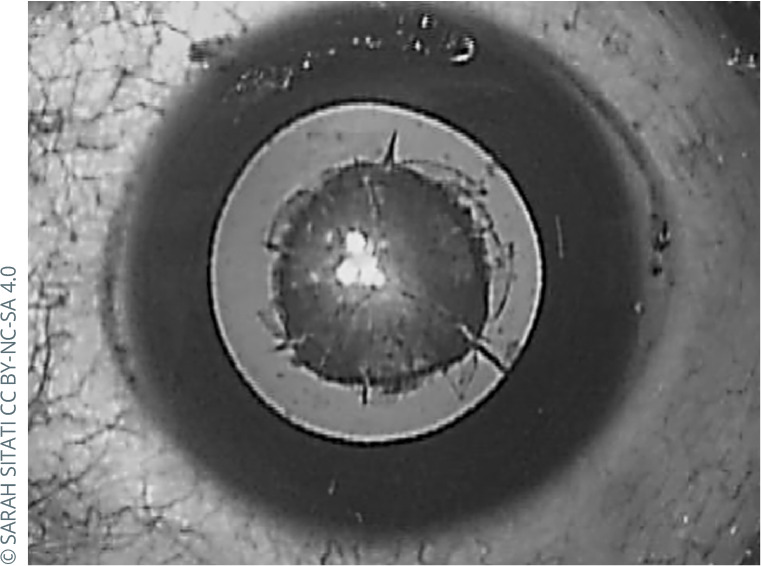
Dense central cataract of more than 4 mm in diameter. kenya

Examine the eyes for **signs which would make surgery more difficult (Figure 1)**, such as microphthalmos (small eyes), a central corneal opacity, iris coloboma (“cat's eye”), or signs of chronic inflammation (bound down, irregular pupils).

Examine the lens through an undilated pupil to assess the location and density of the lens opacities. Estimate the diameter of the opacity (Figure 1). If the peripheral part of the lens is clear (as in nuclear and posterior subcapsular cataract), try to examine the retina and optic nerve around the opacity using an indirect ophthalmoscope. Retinoscopy can also give useful information about the visual axis.

**Figure 2:**
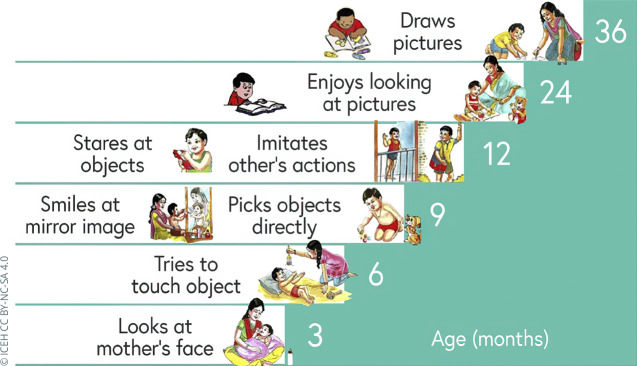
Visual milestones for children aged 3–36 months

**Figure 3:**
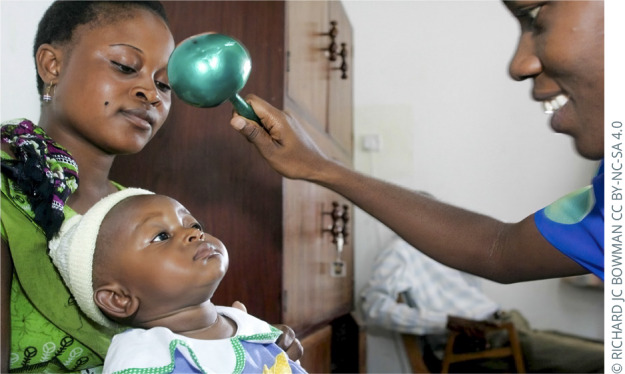
Using a bright object to check fixation and following in a baby. tanzania

It is important to assess visual function, as the level of vision is a major factor when deciding whether surgery should be performed straight away or whether it can be postponed. Measuring visual acuity is very difficult in young children, and the vision milestones outlined in Figure 2 could be useful. You can also use a brightly coloured object to check the child's fixation and following (Figure 3).

### Systemic examination

The purpose of the systemic examination is to identify clinical signs which may suggest an underlying cause, and to assess whether the child is fit for anaesthesia. If there are there are concerns that a systemic condition is present, such as developmental delay, microcephaly, cardiac deficits, or hearing anomalies, examination by a paediatrician is indicated to assess fitness for anaesthesia. Echocardiography should be undertaken, if available, when congenital rubella syndrome is suspected. Laboratory tests may be needed, or genetic testing. It is important to note that congenital rubella syndrome can only be confirmed by raised rubella-specific IgM (not IgG) during the first year of life. After that, raised antibodies may be due to acquired rubella.

## Decision making

After examining the child, is it important to decide:

1. Whether surgery needs to be performed straight away
2. Whether the child is fit for general anaesthesia.

It is important to bear in mind that cataract surgery and general or ketamine anaesthesia in young children are not without risks, optical correction after cataract extraction can be challenging, and regular follow-up is essential.

Comprehensive examination of children in the clinic may be difficult. However, because of the risks involved, examination under anaesthesia should be avoided unless done on the day of surgery.

### Is surgery needed immediately?

Findings which mean that surgery should be performed straight away (as long the child is fit for anaesthesia), or could be postponed, are shown in Table 1.

**Table 1:** Can congenital cataract surgery be postponed? Findings to guide decision making.

Immediate surgery	Surgery can be postponed
Significant vision impairment or the child has not reached their visual milestones	Visual milestones for their age have been met
Nystagmus is present	No nystagmus
The cataracts are total, or affect 3–4 mm or more of the central part of the lens	The cataracts measure less than 3–4 mm
No, or a very poor, view of the retina	The view of the retina is good or reasonably good
	The parents can be relied upon to bring their child back for review

## Management in the eye department

In high-income settings, babies diagnosed with dense bilateral cataract shortly after birth are not operated on until they are 6 weeks old. Surgery is somewhat easier at this age, and there are fewer short- and longer-term complications of surgery, particularly glaucoma.

Surgery is only the start of the care these children need; regular, long-term follow-up is essential to detect and manage complications and to ensure that children have the best optical correction. Frequent counselling of parents is required so that they understand their children's need for life-long care.

### Non-surgical management

Conservative management is generally indicated only for small, partial cataracts that do not affect vision. Conservative management includes spectacle correction, patching, and dilation therapy to manage amblyopia. Follow-up is required to detect worsening of the signs and symptoms.

### Surgical management

Surgery is the only treatment for significant bilateral cataracts. Lens wash-out, combined with posterior capsulotomy and anterior vitrectomy, is the preferred procedure. The posterior capsule and anterior vitreous face should only be left intact in children aged over the age of five if good follow-up can be guaranteed and a YAG laser is available. Capsulotomy with anterior vitrectomy can be done from an anterior approach prior to intraocular lenses implantation or a posterior pars plana approach after IOL implantation into an intact capsular bag. Intracameral antibiotics may protect against postoperative endophthalmitis. Wilson et al have published a freely available and useful review on surgical techniques and which power intraocular lens to use.^3^

Children may not return promptly for surgery on their second eye, which can then become densely amblyopic. For this reason, surgery on both eyes during the same admission (note: not at the same time) is recommended. This also reduces costs and inconvenience for parents.

### Options for immediate correction of aphakia in children

- Intraocular lenses can be inserted during surgery in children if the corneal diameter is greater than 9 mm. The target refraction is hypermetropic, based on age, to take account of changes in refractive error as the eyes grow.^3^ Biometry in children has to be done at the time of surgery. In high-income countries where contact lenses and glasses are readily accessible and replaceable, most surgeons do not insert an IOL below the age of 18 months, but in some low-income settings where follow up is poor, IOLs may be the best option for younger children.
- Contact lenses can be used, if available, for infants under the age of one and in microphthalmic eyes. Use may be limited by cost, as the lenses need to be changed frequently as the eyes grow. Parents need to accept them, and be able to insert, remove, and clean the lenses.
- Aphakic glasses are a safe and cost-effective method, but they require paediatric frames and careful dispensing. They are unsightly and heavy, and keeping them in position can be difficult. Loss and breakage are also big problems in settings where access to eye care and resources are limited.

If a child has been left aphakic, secondary intraocular lens implantation at any age over one year is possible if there are problems with contact lenses or glasses.

## Postoperative complications

Short-term postoperative complications include uveitis, corneal haze, dislocation of the intraocular lens, and wound leakage with or without iris prolapse. Removal of non-absorbable corneal sutures is important to prevent corneal suture abscesses.

Longer term complications include opacification of the visual axis, which can be treated using YAG laser or surgically. Chronic glaucoma occurs in 10–25% of children following cataract surgery; the younger the age at surgery, the greater the risk. Glaucoma can be managed with topical medication, cyclodiode treatment, or angle surgery.

**Figure:**
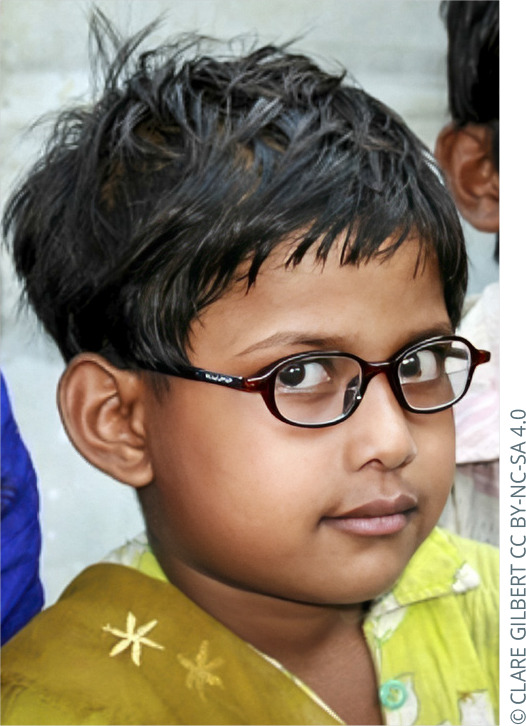
Child wearing executive bifocal spectacles after cataract surgery. bangladesh

## Amblyopia

Amblyopia is the most common cause of low vision in young children after cataract surgery. This can be minimised by the following:

- **Early surgery**, particularly for congenital cataract
- **Accurate refractive correction** after surgery. Even in children with an intraocular lens, their refractive error will change with eye growth and spectacle correction will be required at times. Regular postoperative follow-up, with refraction and new spectacles when necessary, are essential. For children under 5 years of age, single vision spectacles corrected for near vision (a +3.00 add on top of cycloplegic refraction) are needed because because they tend to interact with objects and people close to them. Bifocals are needed for children aged 5 years and above.
- **Intensive occlusion therapy** is essential after unilateral cataract surgery; it may be useful in children with bilateral cataracts if one eye is more amblyopic than the other.
- **Ensuring the visual axis stays clear.** Even if primary capsulotomy and anterior vitrectomy have been performed, secondary opacification is common, particularly in children with congenital cataract.

Spectacle correction and occlusion therapy, combined with frequent re-examination and acuity testing, are important. Amblyopia is challenging to manage in children who present late.

**“Amblyopia is the most common cause of low vision in young children after cataract surgery.”**

## Prevention

Rubella immunisation is now included in many national immunisation programmes, which should reduce congenital rubella syndrome. Treatment of eye or general conditions with topical or systemic steroids should be kept to a minimum, both in terms of the dose and the duration of treatment. Early detection, combined with high quality eye care and follow-up, maximise the chance of a good visual outcome.

The World Health Organization recommends that there is one tertiary level child eye care centre with a well trained and equipped team for every 10 million population. Counsellors and experienced optometrists are very important members of the team.

**Useful videos**

CyberSight Webinar: Surgical management of congenital cataracts www.youtube.com/watch?v=OEcya5f8ssA

Paediatric surgery in Lahan, Nepal www.youtube.com/watch?v=QzIVy8bcnag
